# Fully Automatic Artificial Intelligence Liver Anatomy Segmentation in the Management of Colorectal Liver Metastases

**DOI:** 10.7759/cureus.86072

**Published:** 2025-06-15

**Authors:** Andrea Chierici, Lisa Guzzi, Sebastien Goffart, Nizar Kamoun, Manuel Gargiulo, Patrick Chevallier, Antonio Iannelli, Rodolphe Anty, Hervé Delingette, Fabien Lareyre, Juliette Raffort

**Affiliations:** 1 Digestive Surgery, Centre Hospitalier Universitaire de Nice, Nice, FRA; 2 National Institute for Research in Digital Science and Technology (INRIA), Epione Team, Université Côte d'Azur, Sophia Antipolis, FRA; 3 Radiology, Centre Hospitalier Universitaire de Nice, Nice, FRA; 4 Hepatology, Centre Hospitalier Universitaire de Nice, Nice, FRA; 5 Vascular Surgery, Centre Hospitalier d'Antibes Juan-les-Pins, Antibes, FRA; 6 Clinical Biochemistry, Centre Hospitalier Universitaire de Nice, Nice, FRA

**Keywords:** colorectal cancer liver metastases, deep learning artificial intelligence, hepato-biliary-pancreatic surgery, nnu-net, oncological general surgery

## Abstract

Introduction: Artificial intelligence is gaining increasing interest in medical image segmentation, including liver cancer. However, the literature lacks model implementation in the setting of colorectal liver metastases for treatment planning.

Materials and methods: We collected the portal phase abdominal CT scan images from the Nice University Hospital hepatobiliary oncologic multidisciplinary discussion of 80 patients with colorectal liver metastases, before treatment. Data from 70 patients was exploited to train and test the nnU-Net model to automatically perform parenchyma, portal vein, hepatic veins, cava vein, and colorectal liver metastases segmentation. Data from the remaining 10 patients was used for external validation.

Results: The Dice score for parenchyma segmentation was 0,964 and 0,955 in the test and validation dataset, respectively. For portal vein segmentation, a centerline Dice (clDice) of 0,758 and 0,736 was highlighted, while for hepatic veins it resulted to be 0,758 and 0,577. Cava vein segmentation showed a clDice of 0,805 and 0,734. Concerning colorectal liver metastases, the Dice score was 0,693 and 0,61.

Conclusion: The nnU-Net showed promising segmentation results, especially for liver parenchyma. Its task could be useful to help physicians decide which is the best treatment strategy based on individual anatomical characteristics and disease extension. Training the model on a larger dataset with the same characteristics could help improve segmentation performances.

## Introduction

Colorectal cancer (CRC) is a significant global health concern, ranking as the third most common cancer and the second leading cause of cancer-related mortality worldwide. Annually, 20 million new cancer cases are diagnosed, with CRC accounting for 9.6%; and 9.7 million cancer-related deaths occur, with 9.3% linked to CRC. Southern Europe reports the highest incidence rates (25.1/100,000 males and 16.6/100,000 females), while Middle Africa has the lowest (2.4/100,000 males and 2.2/100,000 females) [1]. By 2040, CRC incidence is projected to increase by 63%, with 3.2 million new cases and a 73.4% rise in mortality [2].

Approximately 20% of CRC diagnoses involve synchronous metastatic disease, with another 25% of initially localized cases developing distant metastases. The liver is the most common site for metastases (62% in colonic cancer and 61% in rectal cancer), followed by the thorax and peritoneum [3,4].

Advancements in diagnostic and treatment modalities have drastically improved the prognosis of patients with colorectal liver metastases (CRLM). While CRLM was once managed palliatively, multidisciplinary approaches now allow personalized treatment plans. Five-year survival rates have improved significantly, with resectable CRLM cases achieving 35-65% survival and initially unresectable cases achieving 33% survival following systemic therapy and downstaging [5,6]. Accurate imaging, including CT, MRI, and ultrasonography, is vital for assessing disease extent, treatment response, and recurrence, although each modality has limitations in sensitivity and specificity [7-9]. Recent efforts to enhance imaging accuracy have included new techniques and contrast agents, though their clinical applicability remains constrained by complexity and reproducibility challenges [10].

Artificial intelligence (AI) is increasingly recognized for its potential in medical imaging. Early computer-aided diagnosis (CAD) systems have evolved with the advent of machine learning and deep learning (DL), particularly using deep convolutional neural networks (DCNNs) for image analysis. DCNNs process input data through convolutional and pooling layers to extract features, classify tissues, and segment anatomical structures. These models have demonstrated success in detecting various cancers, including breast, lung, colorectal, and liver cancers [11-14]. Despite promising initial results for CRLM detection and liver segmentation, challenges remain in data availability, methodological standardization, and generalizability of findings [15,16]. AI’s role in CRLM management is still developing, requiring further research to fully realize its potential. Among DCNN models, nnU-Net [17] has shown promising results in multiple organ automatic segmentation [18]. Concerning the liver, research has been conducted to evaluate this model's performance in parenchyma and tumor segmentation showing high performance [19,20]. However, the use of nnU-Net in clinical practice is still limited since the literature lacks studies evaluating its accuracy in producing full anatomical liver parenchyma, vessels, and tumor segmentation that could be used alone for treatment planning.

The aim of this study is to evaluate the performance of the DCNN nnU-Net algorithm, an implementation of the U-Net model [21], in liver segmentation along with its venous (portal, hepatic, and cava) structures and CRLMs on a CT scan portal phase database issued from a real-world setting.

Our hypothesis is that nnU-Net could automatically diagnose CRLMs from portal-phase CT scans of CRC patients, providing both segmentation and a spatial representation of CRLMs distribution along with the main anatomical features, to help management and treatment decisions.

## Materials and methods

We retrospectively analyzed the report of the weekly multidisciplinary team meeting for liver cancer at the University Hospital of Nice, which is a tertiary referral center for liver cancer management, from October 1, 2022, to February 28, 2024. The multidisciplinary team is composed of at least one hepatologist, one oncologist, one radiologist, one radiation therapist, one liver surgeon, one oncology nurse, and one secretary. During the weekly meeting, each patient’s file which is managed for liver cancer in this center is discussed at the moment of diagnosis and whenever a clinician involved in the patient's care deems that the present treatment should be rediscussed with the other healthcare figures (e.g.: after new imaging tests, clinical worsening, etc.).

Due to the retrospective nature of data collection and analysis, the dataset was recorded in the institutional register but IRB was deemed unnecessary and patients were informed of the inclusion of their anonymized data in the current study.

The primary endpoint of this study is to compare the liver anatomical structures and CRLM segmentation performance of nnU-net compared to manual segmentation. The train, test, and validation dataset is issued from the multidisciplinary oncologic hepatobiliary discussion register of the University Hospital of Nice with the aim of developing a model with data coming from patients managed and treated for CRLM, rather than from publicly available standard imaging datasets, to be used on patients in the same setting for everyday clinical decision making.

Inclusion and exclusion criteria

Inclusion criteria were patients diagnosed with CRLM, who did not receive any prior intervention, and for whom a portal phase, abdominal CT scan was available. Only CT scans performed at the moment of diagnosis and before any treatment directed to CRLM were included.

Patients who had liver cancer other than CRLM were excluded. Furthermore, patients with CRLMs who already had been treated with an interventional radiology procedure (radiofrequency or microwave ablation, transarterial chemoembolization, transarterial radioembolization, etc.), a surgical operation, or a systemic treatment, were excluded.

nnU-Net training and testing

The abdominal contrast-enhanced CT scans of the included patients were retrieved, and the portal phase was selected to undergo the manual segmentation process. Manual segmentation was performed with the 3D Slicer software, version 5.6.2 (Earth, TX, USA) [22]. Segmentation included liver parenchyma, portal veins, hepatic veins, cava veins, and CRLMs.

Before proceeding to model training and testing, CT scan portal phase volumes underwent preprocessing to adapt to nnU-Net elaboration. Firstly, imaging volumes went through Z-score normalization for standardization. After that, we performed resampling and resizing to obtain 3D low-resolution volumes with a 512x512x128 fixed shape. No data augmentation was carried out to enhance dataset size.

**Figure 1:**
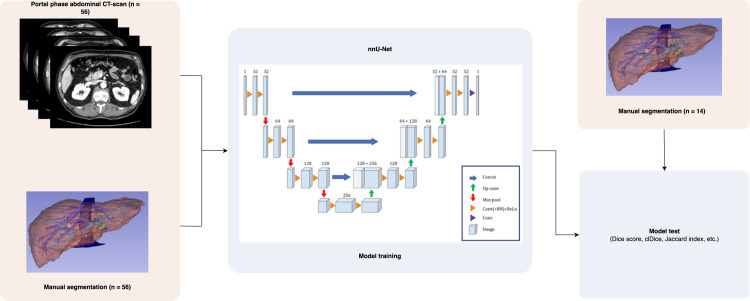
Flow-chart depicting the training and test process of nnU-Net. Created by the authors for this publication.

Then, data was randomly split to obtain a training dataset and a test dataset with an 80% to 20% ratio. Then, the nnU-Net model was trained exploiting the training dataset along with the pixel labels coming from the manual segmentation. Once trained, the nnU-Net model was used to produce fully automatic CT scan segmentation on the test dataset, and this was compared with manual segmentation previously performed on the same CT scans. The whole process is outlined in Figure 1. Model training and testing were performed with Python 3.10 (Python Software Foundation, Wilmington, DE, USA) with the environment package PyTorch [23]. 

U-Net [21] is a DCNN made up of 23 convolutional layers. Its name comes from its architecture, depicted in Figure 1, which consists of a contracting and an expansive path, also known as the encoder and the decoder parts of the model. The encoder identifies relevant features in the input image. While capturing contextual information, the encoder also reduces the spatial resolution of the input two-fold while doubling the feature channels at each layer. In the expansive path, data is decoded, and the input is progressively restored to the initial resolution while the features identified by the encoder are located. Skip connections between encoder and decoder layers processing input of the same resolution are added to preserve the spatial information for the expansive path that are lost in the contracting path. This helps the decoder locate features more accurately.

The nnU-Net implementation allows automatic hyperparameter settings based on given data [17]. In a neural network, hyperparameters are the variables that determine the network structure (number of layers, activation functions, etc.) and how the network is trained (batch size, learning rate, number of epochs, etc.) and in this case, are not set manually before training but automatically depending on the training dataset characteristics. 

Performance measures

To evaluate the nnU-Net model's accuracy in segmenting liver parenchyma, portal vein, hepatic veins, cava vein, and CRLMs, manual segmentation served as the reference standard. The following metrics were used to assess segmentation performance [24,25]: (i) True Positives (TP): Pixels correctly labeled as belonging to a specific structure (e.g., liver parenchyma); (ii) True Negatives (TN): Pixels correctly identified as not belonging to a structure (e.g., veins or CRLMs); (iii) False Positives (FP): Pixels incorrectly assigned to a structure; (iv) False Negatives (FN): Pixels mistakenly identified as not belonging to a structure.

Key Performance Measures

Precision: Proportion of correctly segmented pixels, calculated as TP/(TP+FP).

Recall: Proportion of actual structure pixels correctly identified, calculated as TP/(TP+FN).

False positive rate (FPR): Ratio of pixels falsely assigned to a label, calculated as FP/(FP+TN).

False negative rate (FNR): Probability of misclassifying pixels belonging to a label, calculated as FN/(FN+TP).

Overlap Metrics

Dice score: Measures overlap between predicted and ground truth segmentations, calculated as 2TP/(2TP+FP+FN). Ranges from 0 (no overlap) to 1 (perfect overlap).

Jaccard index: Measures similarity between segmentations, calculated as TP/(TP+FP+FN). Ranges from 0 to 1.

Geometric Metrics

Hausdorff distance (HD): Measures the largest distance between points in the predicted and ground truth segmentations, with the 95% HD reducing outlier influence.

Tubular Structure and Topology Metrics

Centerline dice score (clDice): Evaluates overlap for tubular structures, focusing on segmentation masks and morphological skeletons [26].

Betti numbers (b0, b1): Represent the number of connected components (b0) and holes (b1) in the segmentation [27].

Euler number: Difference between b0 and b1, reflecting segmentation topology [28].

These metrics comprehensively assess segmentation accuracy, overlap, geometry, and topology for the nnU-Net model.

External validation

We retrospectively gathered additional contrast-enhanced CT scans, with the same inclusion process reported for nnU-Net training and testing, from the report of the weekly multidisciplinary team meeting for liver cancer at the University Hospital of Nice from July 1, 2021, to December 31, 2021. A registrar radiologist performed manual liver, vascular (portal vein, hepatic veins, cava vein), and CRLMs segmentation under a senior consultant radiologist's supervision. The trained nnU-Net model was exploited to perform automatic segmentation which was compared with the same accuracy measures to the radiologists’ manual segmentation.

## Results

Patients and CT scan characteristics

**Figure 2:**
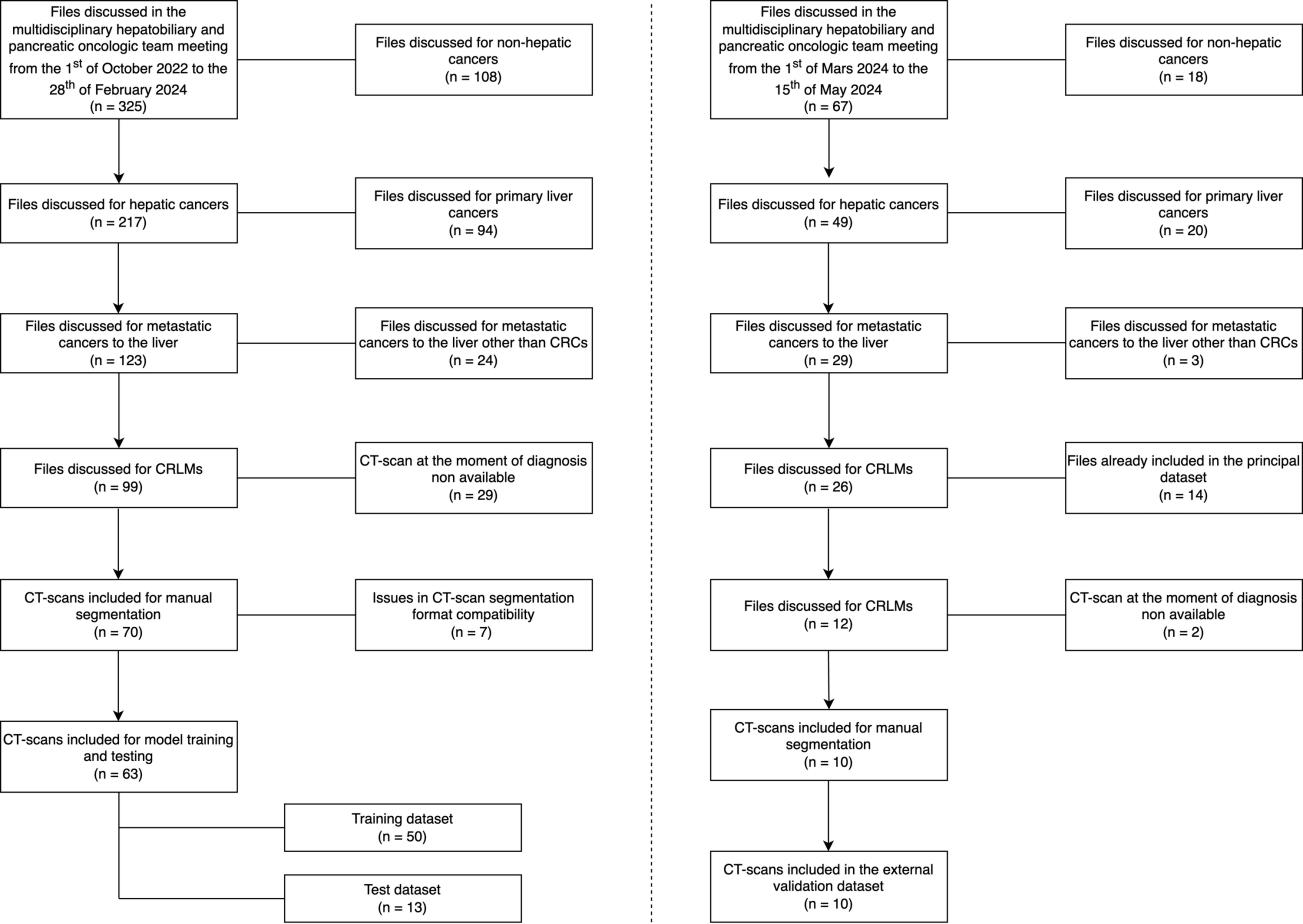
Flow-chart depicting the inclusion process. CRC: colorectal cancer; CRLM: colorectal liver metastases.

**Figure 3:**
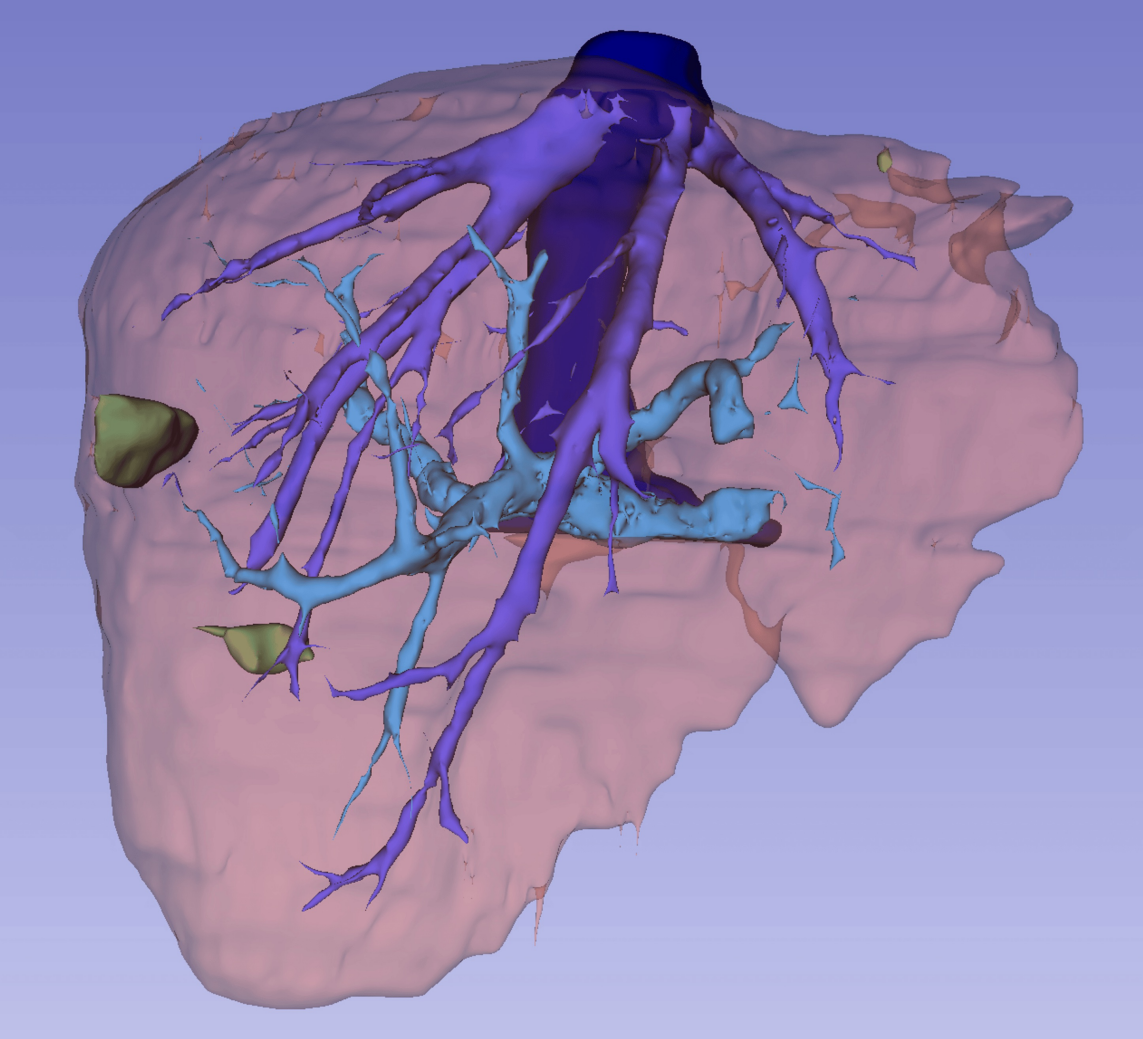
Ground truth of liver parenchyma (brown), portal vein (light blue), hepatic veins (blue), cava vein (dark blue), and CRLMs (green). CRLM: colorectal liver metastases.

In total, 70 portal phase contrast-enhanced CT scans were included, and manual segmentation of liver parenchyma, portal vein, hepatic veins, cava vein, and CRLMs was performed. A flowchart depicting the inclusion process is shown in Figure 2 and an example of manual segmentation is reported in Figure 3. Two hundred and sixty-two patients were excluded based on the above-cited criteria.

The characteristics of the included patients are detailed in Table 1.

**Table 1:** Baseline characteristics of the 70 included patients and CT scans. Continuous variables are expressed as median (interquartile ranges). Categorical variables are expressed as n (%). CRLM: colorectal liver metastases.

Variables	n (%)
Age, y	63 (52 – 71)
Gender, male	35 (50)
Colonic cancer	62 (89)
Ascending colon	15 (21)
Transverse colon	1 (2)
Descending colon	29 (41)
Sigmoid colon	16 (23)
Rectal cancer	8 (11)
Synchronous CRLM	54 (77)
Multiple CRLM	53 (76)
Bilobar CRLM	29 (41)
CRLMs for patient	5 ± 6
CT scan	
Our institution	44 (63)
Slices thickness	
1 mm thickness	62 (89)
3 mm thickness	6 (9)
5 mm thickness	1 (2)

Fifty-four (77%) patients were diagnosed with synchronous CRLM while the other 16 (23%) were diagnosed with de-novo CRLMs during CRC follow-up. Most of the patients (53, 76%) presented multiple CRLMs and in 29 (41%) cases CRLMs were located in both liver lobes.

Concerning CT scans, 44 (63%) were performed in the Radiology Department of the University Hospital of Nice while the others were realized in other hospitals where the initial diagnosis of CRLMs was made and then imported into our institutional radiology database for multidisciplinary discussion. Sixty-two (89%) CT scans had 1 mm slice thickness, six (9%) 3 mm slice thickness, and one (2%) 5 mm slice thickness.

For the external validation dataset, 10 additional patients were included with the same criteria. Their baseline characteristics are detailed in Table 2. No significant difference was found between the groups' baseline characteristics.

**Table 2:** Baseline characteristics of the 10 patients of the external validation dataset. Continuous variables are expressed as median (interquartile ranges). Categorical variables are expressed as n (%). CRLM: colorectal liver metastases.

Variables	n (%)
Age, y	69 (64 – 72)
Gender, male	6 (60)
Colonic cancer	8 (80)
Ascending colon	0 (0)
Transverse colon	1 (10)
Descending colon	3 (30)
Sigmoid colon	4 (40)
Rectal cancer	2 (20)
Synchronous CRLM	7 (70)
Multiple CRLM	8 (80)
Bilobar CRLM	5 (50)
CRLMs for patient	4 ± 9
CT scan	
Our institution	3 (30)
Slice thickness	
1 mm thickness	8 (80)
3 mm thickness	2 (20)
5 mm thickness	0 (0)

Automatic segmentation results

The results of automatic segmentation performed through nnU-Net are fully detailed in Table 3.

**Table 3:** Performance measures for automatic segmentation with nnU-Net. FPR: false positive rate; FNR: false negative rate; HD: Hausdorff distance; clDice: centerline Dice; CRLM: colorectal cancer metastases.

Metrics	Liver parenchyma	Portal vein	Hepatic veins	Cava vein	CRLM
Dice score	0.964 ± 0.004	0.759 ± 0.076	0.795 ± 0.059	0.812 ± 0.117	0.693 ± 0.292
Jaccard index	0.931 ± 0.008	0.618 ± 0.101	0.663 ± 0.081	0.696 ± 0.14	0.585 ± 0.268
Precision	0.963 ± 0.012	0.809 ± 0.079	0.836 ± 0.1	0.835 ± 0.135	0.702 ± 0.285
Recall	0.966 ± 0.006	0.732 ± 0.131	0.782 ± 0.131	0.802 ± 0.13	0.707 ± 0.317
FPR	0.001 ± 0.001	0.001± 0.001	0.001 ± 0.001	0.001 ± 0.001	0.001 ± 0.001
FNR	0.034 ± 0.006	0.268 ± 0.131	0.218 ± 0.131	0.198 ± 0.13	0.29 ± 0.32
HD 95%	4.29 ± 1.023	8.35 ± 4.42	6.95 ± 5.79	9.64 ± 7.34	59.8 ± 62.59
clDice	0.967 ± 0.016	0.758 ± 0.078	0.762 ± 0.08	0.805 ± 0.157	0.677 ± 0.293
b0	6.75 ± 3.1	15.31 ± 14.23	26.08 ± 17.07	0.31 ± 1.11	2.15 ± 2.08
b1	18 ± 15.2	19.08 ± 28.87	31.92 ± 55.03	0.38 ± 0.96	1.54 ± 4.67
Euler	12.5 ± 13.8	34.23 ± 32.98	56.62 ± 61.7	0.69 ± 1.38	3.69 ± 4.75

For liver parenchyma, the mean Dice score was 0.964 ± 0.004 and the Jaccard index was 0.931 ± 0.008.

Portal vein segmentation was performed by nnU-Net with a Dice score of 0.76 ± 0.076 and a Jaccard index of 0.618 ± 0.101. The mean clDice was 0.758 ± 0.078.

Hepatic veins automatic segmentation was performed with a 0.795 ± 0.059 Dice score and a Jaccard index of 0.663 ± 0.081. The mean clDice was 0.762 ± 0.080.

For the cava vein, the mean Dice score was 0.812 ± 0.117 and the Jaccard index was 0.696 ± 0.140, while clDice was 0.805 ± 0.157.

Finally, for CRLMs, the Dice score was 0.693 ± 0.292 and the Jaccard index was 0.585 ± 0.268. Betti’s numbers were 2.15 ± 2.08 (b0) and 1.54 ± 4.67 (b1).

External validation

Model performance in producing liver parenchyma segmentation on the external validation dataset was characterized by a Dice score of 0.96 ± 0.02 and a Jaccard index of 0.91 ± 0.02.

Concerning portal vein segmentation, the Dice score was 0.71 ± 0.15, the Jaccard index was 0.56 ± 0.17, and clDice was 0.74 ± 0.14.

For hepatic veins segmentation, instead, we highlighted a Dice score of 0.57 ± 0.16, a Jaccard index of 0.42 ± 0.16, and a clDice of 0.58 ± 0.15.

Cava vein segmentation performance resulted in a Dice score of 0.62 ± 0.22, a Jaccard index of 0.48 ± 0.2, and a clDice of 0.73 ± 0.31.

In conclusion, for CRLMs, a Dice score of 0.61 ± 0.33, a Jaccard index of 0.51 ± 0.33, and Betti numbers of 6.89 ± 12.34 (b0) and 1 ± 1.5 (b1) were highlighted.

The results of external validation are summarized in Table 4.

**Table 4:** Performance measures for automatic segmentation with nnU-Net on the external validation dataset. FPR: false positive rate; FNR: false negative rate; HD: Hausdorff distance; clDice: centerline Dice; CRLM: colorectal cancer metastases.

Metrics	Liver parenchyma	Portal vein	Hepatic veins	Cava vein	CRLM
Dice score	0.955 ± 0.014	0.707 ± 0.147	0.571 ± 0.165	0.619 ± 0.224	0.61 ± 0.336
Jaccard index	0.914 ± 0.025	0.564 ± 0.17	0.416 ± 0.161	0.476 ± 0.202	0.509 ± 0.328
Precision	0.957 ± 0.029	0.763 ± 0.096	0.594 ± 0.21	0.508 ± 0.221	0.643 ± 0.296
Recall	0.953 ± 0.017	0.689 ± 0.212	0.581 ± 0.192	0.891 ± 0.035	0.699 ± 0.314
FPR	0.001 ± 0.001	0.001 ± 0.001	0.001 ± 0.001	0.001 ± 0.001	0.001 ± 0.001
FNR	0.047 ± 0.017	0.311 ± 0.212	0.419 ± 0.192	0.109 ± 0.035	0.3 ± 0.314
HD 95%	6.3 ± 5.21	8.42 ± 10.01	26.22 ± 22.67	20.3 ± 20.5	23.97 ± 21.93
clDice	0.934 ± 0.043	0.736 ± 0.135	0.577 ± 0.153	0.734 ± 0.308	0.627 ± 0.388
b0	9.77 ± 7.20	19.44 ± 9.98	39.56 ± 16.3	2.67 ± 6.89	6.89 ± 12.34
b1	116.1 ± 292.3	50.67 ± 55.59	27.22 ± 28.03	0.22 ± 0.44	1 ± 1.5
Euler	111 ± 287.4	63 ± 64.88	62.11 ± 42.63	2.89 ± 6.83	7.22 ± 13.73

## Discussion

Our research achieved impressive accuracy in liver parenchyma segmentation using nnU-Net. Dice score, Jaccard index, precision, and recall all exceeded 0.9, indicating near-complete overlap between automatic and manual segmentations. A mean HD 95% of 4.29 reflects minimal edge distance between predicted and ground truth segmentations. Low FPR (0.0005) and FNR (0.034) confirm accurate pixel classification. External validation produced comparable results, reaffirming the model's reliability.

For vascular structures, nnU-Net delivered positive but slightly lower performance. Portal vein and hepatic veins segmentation showed clDice scores of 0.758 and 0.762, indicating high topology similarity. Cava vein segmentation achieved a clDice of 0.805, with precision and recall values of 0.809 and 0.732, 0.836 and 0.782, and 0.835 and 0.802 for the portal vein, hepatic veins, and cava vein, respectively. External validation yielded consistent results only for the portal vein (clDice = 0.74), likely due to training with portal phase images, which limited hepatic and cava vein enhancement.

CRLM segmentation also showed promise, with a Dice score of 0.693 ± 0.292, indicating good overlap despite variability across CT scans. Betti numbers revealed a mean misidentification of 2 ± 2 CRLMs per scan, from a dataset average of 5 ± 6 lesions per patient. The FNR of 0.29 ± 0.32 reflects moderate misclassification, varying significantly among patients. External validation showed slightly reduced performance (Dice score = 0.61) and increased variability in lesion identification (b0 = 7 ± 12). Despite these challenges, nnU-Net demonstrated strong potential for accurate anatomical segmentation.

With a limited and heterogeneous dataset, nnU-Net could almost perfectly segment liver parenchyma, and this was confirmed in the external validation process. We also obtained an encouraging performance for vascular and CRLMs segmentation, but the results were substantially confirmed only for portal vein segmentation and CRLMs, while cava vein and hepatic veins automatic segmentation performance where inferior on the external validation dataset.

The development of AI-dependent tools to improve cancer diagnosis and treatment has become a central topic in recent literature. The application of AI and especially DCNN sweeps from histologic to any kind of radiologic image as long as a digital picture is available to train an AI model. Concerning pathologic images, efforts have been made to train DCNNs to identify CRLMs, distinguish them from other malignant and benign lesions, and predict patients’ prognosis [29-32].

In a few recent cases, research has been conducted to exploit radiologic digitalized images to diagnose, segment, or eventually distinguish CRLM from other liver lesions. Kim et al. [33] trained the YOLOv3 [34], a DCNN object detection model, on 4386 slices of pre-treatment portal phase CT scan of CRLM, cysts, or hemangiomas. Ground truth was defined by manual identification of the area of the lesions and annotation by a resident and an experienced radiologist. On the validation dataset, DCNN diagnostic performance was 87.5% sensitivity, comparable to radiologists, and 22.2% specificity, which was significantly inferior to those of radiologists. Focusing principally but non exclusively on malignant liver lesions, Midya et al. [35] similarly trained a DCNN, the Inception v3 network [36], to classify hepatocellular carcinoma, intrahepatic cholangiocarcinoma, CRLMs, and benign liver tumors on portal phase CT-scans. Training and testing were done on 814 pre-treatment CT scans performed with the same acquisition method. Overall accuracy in correct liver lesion classification was 96.27% and it increased to 98.63% when considering CRLMs only.

With regard to CRLM segmentation, two studies focused on the performance of DCNN models [37,38]. Vorontsov et al. [37] trained and tested a full CNN, whose architecture was inspired by U-Net, on the publicly available Liver Tumor Segmentation Benchmark (LiTS) dataset [39]. External validation was then performed on 26 portal phase CT scans from the authors’ tertiary referral center. Dice score varied from 0.14 to 0.68 depending on the size of CRLMs (<10 mm and > 20 mm respectively). The performance of this model was inferior to those showed by nnU-Net, although a superiority of this model to the one exploited by Vorontsov et al. cannot be confirmed as substantial differences in the data inclusion process (i.e.: publicly available vs. institutional dataset) exist [37]. However, the most consistent work on automatic CRLM segmentation through DCNN is the one by Wesdorp et al. [38]. The authors exploited 259 portal-phase CT scans of patients with unresectable CRLMs before and/or after systemic treatment. Ground truth was assessed with semi-automatic segmentation manually modified in post-processing by the authors under the supervision of an experienced radiologist. U-Net was then trained and tested separately to produce liver parenchyma and tumor masks to be compared to ground truth. Accuracy measures were reported concerning the test dataset and an external validation cohort showing 0.8 and 0.6 Dice score, 0.75 and 0.69 Jaccard index, 0.89 and 0.85 precision, and 0.84 and 0.78 recall, respectively. These performance metrics on CRLM segmentation were superior to what we obtained on our institutional dataset. This was probably due to larger and highly heterogeneous data and encouraged us to exploit nnU-Net to perform liver anatomical and CRLM segmentation in the same setting.

The automatic segmentation of liver anatomical structures from medical images through AI has also been the center of recent research. Producing an automatic reconstruction of liver anatomy can have several implications, especially related to treatment plans and pedagogy [40-42]. Li et al. developed a DCNN model, whose architecture was inspired by U-Net, on 515 portal phase CT scans to automatically segment liver parenchyma and vessels [43]. For liver parenchyma segmentation the authors obtained a 0.98 Dice score and a 1.52 mm 95% HD, while for portal, hepatic, and cava veins, the Dice score was 0.86, 0.89, and 0.94 respectively. Similarly, Xu et al. elaborated a DCNN model with an encoder-decoder structure on portal phase CT scans from the public 3D-IRCADb-01 and Medical Segmentation Decathlon (MSD) datasets obtaining 0.796 and 0.835 Dice scores for portal and hepatic veins segmentation respectively [44]. They also compared the global Dice score of their model with other DCNN models conceived for segmentation, including U-Net, showing significantly superior performance.

Although AI-driven models in medicine are one of the most popular topics of the moment and new research on the subject is constantly being developed, flaws in study design, methodology, and results report are frequently encountered and DCNN models for CRLMs management are no less. This issue has been addressed specifically for the application of AI to medical imaging by Lareyre et al. who summarized the current guidelines in this field [45]. Moreover, special attention has been dedicated by Müller et al. to the evaluation metrics adopted in this field and their shortcomings [46]. Dice score and Jaccard index are the most appropriate segmentation accuracy measures, with the latter being more sensible to under- or over-segmentation, for polyhedric objects, while clDice has been specifically developed for tubular structures. Sensitivity and specificity are also frequently reported but, due to the large number of pixels annotated, they are less suited than other measures to estimate model performance. On the other hand, accuracy, which is also often used to describe model performance, is not pertinent for this task as it includes TN in the formula, which leads to high scores mostly depending on the fact that the model correctly annotates the background which is usually much bigger than the segmented object. Instead, with respect to object localization, average HD and average 95% HD focus on boundary delineation comparing predicted and ground truth and it is the most adopted measure for this task. 

The robustness of most of the above-mentioned research on CRLM detection and segmentation is affected by the limitations associated with the adopted metrics. Moreover, patient and medical image selection also are sources of bias. Some studies included pre- and post-treatment CT scans from the same patients instead of including images taken at the same time point. Others based their model training and testing on publicly available datasets whose characteristics are far distant from routinely performed CT scans and their volume was often limited. The same can also be said for those studies whose model development is based on CT scans acquired with the same settings. All these factors contribute to a reduced results generalization.

In this research, we used the nnU-Net model for the segmentation of liver parenchyma, vessels, and CRLMs, aiming to develop a comprehensive tool for anatomical reconstruction. Following current guidelines for AI in medical image analysis [47,48], we achieved highly positive results for liver parenchyma segmentation and encouraging outcomes for vessels and CRLMs.

However, the study has limitations. The small number of patients included likely impacted model performance. Compared to similar studies, we trained nnU-Net on a smaller dataset due to the technical challenges of manually segmenting the liver, vessels, and CRLMs. This resulted in a slightly lower overlap between ground truth and predicted segmentation, particularly for CRLMs. A larger dataset could improve model performance.

Additionally, we employed advanced metrics like clDice and Betti numbers to evaluate segmentation quality, focusing on pixel accuracy, object positioning, and identification, particularly for vessels and CRLMs. The heterogeneity of the CT scan dataset used, including variations in acquisition protocols, also influenced model accuracy. Nonetheless, this reflects real-world conditions where radiologic evaluations from various hospitals guide treatment planning. A robust segmentation tool capable of handling diverse imaging protocols could be valuable for clinical use.

Moreover, limiting the analysis to the portal phase impacted hepatic and cava vein segmentation due to lower enhancement compared to the portal vein. However, CRLM follow-up scans rarely include delayed phases, so excluding them aligns with typical clinical practice and maintains model applicability. Despite imaging variability in CRLMs, segmentation performance remained consistent across training and validation datasets. This highlights the potential of nnU-Net for real-world applications.

Finally, a further limitation of our study is the absence of a direct comparison between automated and manual segmentations from multiple expert radiologists. The ground truth annotations were generated by a single radiologist with expertise in hepatic imaging, under supervision, which ensured consistency but did not account for inter-operator variability. In clinical practice, segmentation discrepancies among human experts are common and may influence treatment decisions, especially for small or ill-defined lesions. Including multiple annotations per case would have allowed us to assess the interobserver variability and better contextualize the model’s performance within human-level expectations. Moreover, a formal error analysis, that is, categorizing segmentation failures by anatomical location, lesion size, or contrast quality could further clarify model limitations. While beyond the scope of this study, such analysis represents an important direction for future research, particularly in preparation for clinical deployment.

## Conclusions

In this study, we applied the nnU-Net framework to segment liver parenchyma, major vascular structures, and colorectal liver metastases (CRLMs) from portal phase CT scans obtained in a real-world clinical setting. The model achieved excellent performance in liver parenchyma segmentation, and encouraging, though variable, results for vascular structures and CRLMs. However, external validation revealed reduced consistency, particularly for hepatic and cava veins, and performance for CRLM segmentation was moderate and heterogeneous across cases. These findings highlight the potential of nnU-Net as a foundation for developing fully automated anatomical reconstruction tools, but also underscore the current limitations in generalizability and lesion-specific performance.

The present study should be considered an exploratory step toward clinical integration. Future work must involve larger, multicenter datasets, incorporation of expert-level annotation variability, and a thorough evaluation of workflow integration and resource implications. While nnU-Net shows promise, particularly for liver segmentation, its clinical application will require further optimization and prospective validation to ensure robust, reproducible results across diverse patient populations and imaging protocols.
